# T Cell Dysfunction in Cancer Immunity and Immunotherapy

**DOI:** 10.3389/fimmu.2019.01719

**Published:** 2019-07-19

**Authors:** Anliang Xia, Yan Zhang, Jiang Xu, Tailang Yin, Xiao-Jie Lu

**Affiliations:** ^1^Department of General Surgery, Liver Transplantation Center, The First Affiliated Hospital of Nanjing Medical University, Nanjing, China; ^2^Department of Clinical Laboratory, Renmin Hospital of Wuhan University, Wuhan, China; ^3^Department of Rehabilitation, Huai'an Second People's Hospital, and the Affiliated Huai'an Hospital of Xuzhou Medical University, Huai'an, China; ^4^Reproductive Medicine Center, Renmin Hospital of Wuhan University, Wuhan, China

**Keywords:** T cell dysfunction, immunity, cancer, immunotherapy, tumor microenvironment

## Abstract

In cancer, T cells become dysfunctional owing to persistent antigen exposure. Dysfunctional T cells are characterized by reduced proliferative capacity, decreased effector function, and overexpression of multiple inhibitory receptors. Due to the presence of various inhibitory signals in the complex tumor microenvironment, tumor-specific T cells have distinct dysfunction states. Therapeutic reactivation of tumor-specific T cells has yielded good results in cancer patients. Here, we review the hallmarks of T cell dysfunction in cancer. Also, we discuss the relationship between T cell dysfunction and cancer immunotherapy.

## Introduction

T cells can take part in a variety of immune responses that arise in various diseases, including infection, cancer, autoimmune diseases, and allergic diseases. In acute infections, naive T cells, upon antigen stimulation, are rapidly activated and differentiate into effector T cells (Teff). Teff differentiation involves transcriptional, epigenetic and metabolic reprogramming as well as the acquisition of effector features. After antigen clearance, most Teff die, but a small fraction of them differentiate into memory T cells, which quickly respond when the same antigen reappears (1). Memory T cells downregulate their effector program and own self-renewal ability driven by IL-7 and IL-15 in an antigen-independent manner (2). By contrast, during chronic infections and cancer, the function of T cells becomes compromised, termed T cell dysfunction, due to persistent antigen exposure (3–7). Previous studies have demonstrated that the severity of dysfunction is associated with the level of antigen stimulation (8, 9). Furthermore, specific T cell receptor (TCR)-dependent pathways, for instance, those mediated by nuclear factor of activated T cells (NFAT) and sprouty homolog 2 (SPRY2), have been shown to be involved in T cell dysfunction, in line with the effects of ongoing TCR stimulation (10–12). In addition, chronic antigen stimulation also results in persistent expression of programmed cell death protein 1 (PD-1) by NFAT cytoplasmic 1 (NFATc1) (13). PD-1 may further regulate the level of TCR signaling (14, 15). Therefore, the extent and persistence of antigenic stimulation appear to be vital factors leading to T cell dysfunction and are associated with the severity of dysfunction.

**Figure 1:**
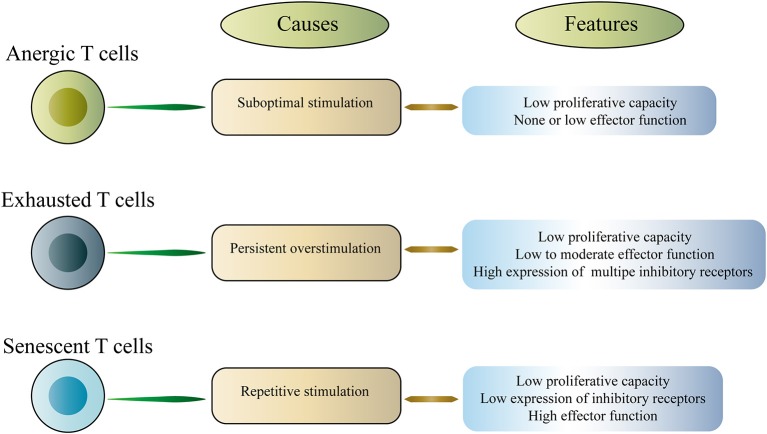
Classification of dysfunctional T cells.

T cell exhaustion is a representative of T cell dysfunction. Exhausted T cells (Tex) differ from other dysfunctional T cells, including anergic T cells and senescent T cells (16–18). Anergic T cells are induced by suboptimal stimulation, whereas senescent T cells enter a terminally differentiated state due to repeated stimulation, which involves irreversible cell cycle arrest and telomere shortening (Figure 1). In this article, we mainly discuss the exhausted T cells. T cell dysfunction was first discovered in mice infected with chronic lymphocytic choriomeningitis virus (LCMV), which is characterized by a progressive loss of function including proliferation, cytokine production and the ability to lyse target cells (3). Subsequently, T cell dysfunction was described in humans with chronic viral infections and cancer (9, 19–21). The acquired dysfunction was related to the co-expression of multiple inhibitory receptors (IRs) including PD-1, cytotoxic T lymphocyte antigen 4 (CTLA-4), T-cell immunoglobulin domain and mucin domain-3 (Tim-3), lymphocyte activation gene 3 (LAG-3), T cell immunoreceptor with Ig and ITIM domains (TIGIT), and others (4, 6, 9, 22–25). Interestingly, studies in mice and humans have shown that dysfunctional CD8^+^ T cells coupregulate multiple IRs, and the type and amount of IRs are associated with the severity of the T cell dysfunction (4, 9, 26, 27). It is worth noting that dysfunctional T cells are not completely useless. Instead, these cells retain a certain level of residual function, and this residual function may be critical *in vivo*, limiting persistent pathogen infection and tumor progression. However, dysfunctional T cells fail to effectively eliminate infection and cancer.

Reversal of T cell dysfunction is becoming more important in improving immunity to cancer. Immune checkpoint blockade, as one modality of cancer immunotherapy, is designed to enhance T cell function and thus exert an effective anti-tumor T cell response (28, 29). One of the major markers of T cell dysfunction is the overexpression of PD-1. Blocking PD-1 or its ligand PD-L1 successfully reactivates T cell function, emphasizing the importance of the PD-1/PD-L1 axis in reversing T cell dysfunction (22). Encouraging results have been observed in a variety of cancer types, such as melanoma, urothelial cancer and non-small-cell lung cancer (NSCLC), with the use of antibodies targeting PD-1/PD-L1 (30–33). However, most patients do not maintain sustained responses to this therapy. The lack of a long-lasting response may be partly explained by the presence of other inhibitory receptors in T cells.

Here, we review the hallmarks of T cell dysfunction in cancer. Also, we discuss the relationship between T cell dysfunction and cancer immunotherapy.

## Traits and Mechanisms of T Cell Dysfunction in Cancer

### Inhibitory Receptors in Dysfunctional T Cells

One of the traits of dysfunctional T cells is the increased and sustained expression of multiple inhibitory receptors, including PD-1, CTLA-4, Tim-3, and LAG-3 (**Figure 3a**). In general, the number of inhibitory receptors expressed by dysfunctional T cells is positively associated with T cell dysfunction. In other words, the greater the number, the more serious the dysfunction. Additionally, functional effector T cells can transiently express inhibitory receptors upon activation. For instance, PD-1 expression rapidly increases after T cell activation and may remain at moderate levels in healthy individuals (34, 35). To control autoreactivity and immunopathology, inhibitory receptors play a key negative regulatory role (36). In healthy adults, for example, circulating PD-1^+^CD8^+^ T cells are not representative of dysfunctional T cells, but instead are representative of effector memory cells (35). In addition, IR expression patterns in dysfunctional T cells also have differences between cancer and infection, albeit with many common features (5). B- and T-lymphocyte attenuator (BTLA) is upregulated in dysfunctional CD8^+^ T cells in cancer, but is absent in dysfunctional CD8^+^ T cells in LCMV (4, 27). This finding shows that the molecular mechanisms that cause IR upregulation and T cell dysfunction may be somewhat different between chronic infections and cancer.

**Figure 2:**
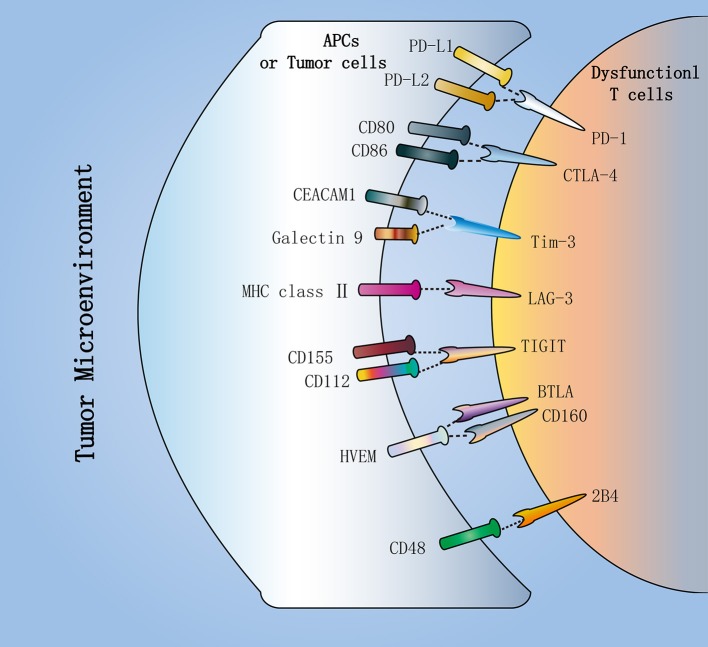
Inhibitory receptors in dysfunctional T cells. Dysfunctional T cells in the tumor microenvironment (TME) express multiple inhibitory receptors, including PD-1, CTLA-4, Tim-3, LAG-3, and TIGIT. They bind to their respective ligands, which are typically expressed by antigen-presenting cells (APCs) or tumor cells in the TME. PD-1, as the major inhibitory receptor, has two ligands PD-L1 and PD-L2. CTLA-4 can compete with the costimulatory molecule CD28 to bind to CD80 and CD86. Additionally, TIGIT can compete with CD226 to bind to CD112 and CD155. In addition, Tim-3 directly binds to Galectin-9 and CEACAM1 to inhibit T cell function. These inhibitory receptors contribute to T cell dysfunction in cancer.

Moreover, each IR binds to its ligand, which is typically expressed by antigen presenting cells (APCs) and tumor cells in the tumor microenvironment (TME), to affect T cell survival, proliferation and function (Figure 2). In view of this, the availability and amount of ligands in the TME is critical for IR to play a negative regulatory role in CD8^+^ T cells. As one of the ligands for PD-1, the expression of PD-L1 in the TME relies on exposure to inflammatory cytokines (37). Conversely, CD155 and CD112, as TIGIT ligands, are continuously expressed in the TME by most melanoma cells and APCs (25). In addition, Tim-3 directly binds to Galectin-9 and CEACAM1 to inhibit T cell function (38, 39). Also, Tim-3 can bind to phosphatidylserine and the DNA binding protein HMGB1 (40, 41).

The molecular mechanisms of IR-mediated immunomodulation of T cells have not been fully elucidated. There are several possible mechanisms to support IR-mediated immune regulation. First, IR competes with costimulatory receptors for binding to its ligand to prevent the formation of microclusters and lipid rafts (25). For instance, CTLA-4 can compete with the costimulatory molecule CD28 to bind to CD80 and CD86 (42), while TIGIT can compete with CD226 to bind to the same ligands CD112 and CD155 (43, 44). The imbalance in the expression levels of IR and costimulatory receptors affects the same ligand binding (45). In patients with metastatic melanoma, CD8^+^ TILs upregulate TIGIT and downregulate CD226, leading to an imbalance of TIGIT/CD226 expression (25). This imbalance may be conducive to inhibiting T cell responses to tumors in the TME, besides T cell inhibition mediated by TIGIT itself (46). Second, activation of T cells by TCR or costimulatory receptors is disrupted by negative signals mediated by PD-1 and CTLA-4 (45, 47). When TIGIT/CD155 is ligated, APCs produce IL-10, which also inhibits T cell function (44). In addition, CD28 can also be expressed by PD-1^+^ T cells. CD28, as a costimulatory molecule, stimulates the activation of naive T cells and promotes cytokine secretion. Recent studies have revealed that the PD-1/PD-L1 axis inhibits T cell function through inactivation of CD28 signaling (48). In a mouse model, conditional knockout of CD28 abolishes the effects of PD-1 blockade (49). This suggests that the CD28/B7 pathway may act as an important role in the efficacy of anti-PD-1 therapy.

Accumulated evidence shows that simultaneous blockade of multiple inhibitory receptors is more effective than single IR blockade in reversing dysfunctional CD8^+^ T cells *in vitro* and *in vivo*. For example, blockade of PD-1 combined with blockade of CTLA-4, Tim-3, LAG-3, or TIGIT blockade, to some extent, can reverse T cell dysfunction and enhance antitumor immunity (23, 25, 26, 50, 51). These inhibitory receptor molecules are derived from different structural families and have different intracellular signaling domains. They can bind to ligands with different expression patterns.

### Inhibitory Cells in the Tumor Microenvironment (TME)

**Figure 3:**
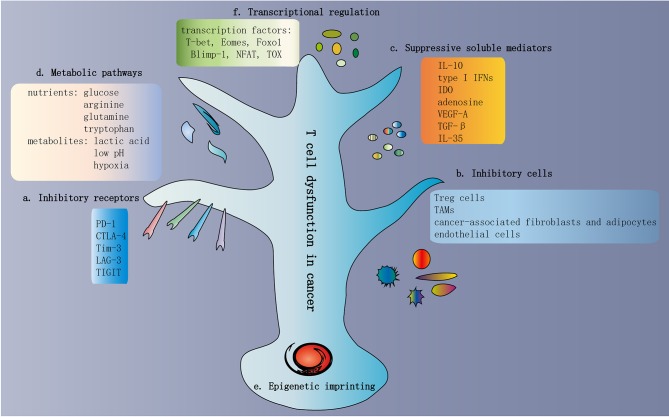
Traits of T cell dysfunction in cancer. **(a)** Inhibitory receptors in dysfunctional T cells. One of the traits of dysfunctional T cells is the increased and sustained expression of multiple inhibitory receptors, including PD-1, CTLA-4, Tim-3, LAG-3, and TIGIT. In general, the greater the number of inhibitory receptors coexpressed by dysfunctional T cells, the more severe the dysfunction. **(b)** Inhibitory cells in the TME. The TME contains various cell types involved in multiple biological processes that promote or inhibit tumor progression. Immunosuppressive cells are present in the TME, which contribute to T cell dysfunction. These inhibitory cells include Treg cells, TAMs, MDSCs, cancer-associated fibroblasts and adipocytes, and endothelial cells. **(c)** Suppressive soluble mediators. Some soluble molecules exist in the TME and mediate T cell dysfunction. These molecules include IL-10, type I IFNs, IDO, adenosine, VEGF-A, TGF-β, and IL-35. **(d)** Metabolic pathways in the TME. The activation of T cells and the exertion of antitumor immunity depend on some common metabolic pathways, such as aerobic glycolysis, amino acid metabolism, glutaminolysis, and *de novo* fatty acid synthesis. These metabolic pathways are also important preconditions for cancer cell proliferation and survival. Hence, within the TME, T cells compete with cancer cells to obtain adequate nutrients. In addition to nutrients, various metabolites are also involved in T cell dysfunction, such as lactic acid, low pH, and hypoxia. **(e)** Epigenetic imprinting of T cell dysfunction. Epigenetic imprinting of dysfunctional T cells differs from that of effector/memory T cells. Persistent PDCD1 demethylation and unique changes in chromatin accessibility occur in dysfunctional T cells. **(f)** Transcriptional regulation of T cell dysfunction. Transcriptional regulation of T cell dysfunction involves changes in the expression patterns and transcriptional connection of some important transcription factors, such as T-bet, Eomes, Foxo1, Blimp-1, NFAT, and TOX. TME, tumor microenvironment; Treg cells, regulatory T cells; TAMs, tumor-associated macrophages; MDSCs, myeloid-derived suppressor cells; IDO, indoleamine 2,3-dioxygenase; TGF-β, transforming growth factor-β.

The TME contains a variety of cell types that participate in various biological processes, which promote or inhibit tumor progression. Immunosuppressive cells are present in the TME and contribute to T cell dysfunction. These inhibitory cells include regulatory T cells (Treg cells), tumor-associated macrophages (TAMs), myeloid-derived suppressor cells (MDSCs), cancer-associated fibroblasts and adipocytes, and endothelial cells (Figure 3b).

Treg cells, as a major group of infiltrating CD4^+^ T cells in the TME, can significantly inhibit the antitumor immunity mediated by T cells (52, 53). Treg cells usually disrupt the activation, proliferation, and survival of effector T cells by producing immunosuppressive molecules, including transforming growth factor-β (TGF-β) and interleukin-10 (IL-10) (6, 54). Notably, multiple IRs are upregulated in highly inhibitory Treg cells, including PD-1, CTLA-4, Tim-3, and TIGIT (55–57). Of course, they also upregulate molecules associated with T cell dysfunction or trafficking, including CCR4, CD39, and CD73, as well as members of the TNF receptor superfamily, such as GITR and OX40 (58–60). Therefore, antibodies targeting CTLA-4, CCR4, and/or GITR on Treg cells can deplete Treg cells, reverse T cell dysfunction, and restore T cell antitumor immunity and immune surveillance on cancer cells (61–63).

TAMs suppress T cell antitumor immunity and promote tumor development, involving functions such as the sustained accumulation of Treg cells and dysregulation of the vasculature due to the expression of chemokines and amino acid-degrading enzymes, such as arginase 1 and indoleamine-2,3-dioxygenase (IDO) (64–66). Similarly, MDSCs enter TME aberrantly, produce nitric oxide and reactive oxygen species, and express arginase 1 and IDO, thereby effectively promoting T cell dysfunction (67, 68). In a mouse model, targeting MDSCs with monoclonal antibodies has been demonstrated to restore the antitumor immune responses and tumor killing ability of tumor-infiltrating T lymphocytes (TILs) (69).

Cancer-associated fibroblasts can secrete cytokines and chemokines, and disrupt the deposition of the extracellular matrix, which shapes the structure of the TME and thus contributes to tumorigenesis (70, 71). T cell dysfunction can also be caused by cancer-associated fibroblasts via the production of TGF-β and vascular endothelial growth factor (VEGF) (72, 73). Moreover, recent findings have also shown that cancer-associated adipocytes impair antitumor immunity and promote tumor malignancy in several cancers (74–76). The mechanism may be mediated by the metabolic and paracrine regulation of tumor infiltrating immune cells and cancer cells.

Endothelial cells may promote T cell dysfunction by improving the production of prostaglandin E2 (PGE2) and CD95L, while impairing T cell recruitment by reducing the expression of vascular cell adhesion molecule 1 (VCAM1) (77–79). The underlying mechanisms of these changes are mediated by hypoxia and VEGF signaling in endothelial cells. In addition, metabolic communication between cancer and endothelial cells, as well as lymphatic endothelial cells, may help impede antitumor T cells and mediate immunosuppression (80–82).

### Suppressive Soluble Mediators

Some soluble molecules are present in the TME that mediate T cell dysfunction. These molecules include IL-10, type I IFNs, IDO, adenosine, VEGF-A, TGF-β, and IL-35 (Figure 3c).

IL-10 is produced by various immune cells and serves as an effective antiinflammatory molecule (83). For instance, natural killer cells, APCs, T cells, and B cells can generate IL-10 (84–87). Interestingly, the dose of IL-10 and the state of T cell activation can affect the effects of IL-10 on T cells *in vivo* (88). On the one hand, IL-10 impairs antitumor immunity and promotes tumor growth in mouse models (89). Simultaneous blockade of PD-1 and IL-10 results in increased survival and delays tumor growth in ovarian cancer, leading to an enhanced antitumor immune response and reduced infiltration of immunosuppressive MDSCs (90). On the other hand, high doses of IL-10 and PEGylated IL-10 hamper the progression of tumors in animals and increase the expansion and function of CD8^+^ TILs expressing elevated IL-10R (88, 91). Thus, IL-10 may have a paradoxical effect on T cells *in vivo*.

Type I IFNs including IFNα and IFNβ may be typical cytokines with a dual role. As key pro-inflammatory cytokines, type I IFNs inhibit viral replication by directly inducing antiviral activity and activating innate immune cells (92). Additionally, type I IFN signaling facilitates the optimal priming of T cells and production of functional effector T cells and memory T cells (93). However, some studies have also shown that IFNα promotes the persistence of viruses in chronic infections and induces T cell dysfunction (94). During the first few days of LCMV infections, blockade of IFNα can effectively prevent the occurrence of T cell dysfunction (94). The mechanism by which IFNα promotes T cell dysfunction may be through the production of immunosuppressive molecules, such as IL-10, PD-L1, and IDO, as well as the stimulation and maintenance of PD-1 expression on T cells (95). Notably, IFNα blockade combined with PD-1 blockade effectively enhances antitumor immune responses in tumor-bearing mice (95).

IDO is expressed by tumor cells, APCs and CD8^+^ T cells in the TME (96–99). IDO activates Tregs by producing kynurenine and inhibits T cell function by depleting the essential amino acid tryptophan (100). In addition, resistance to anti-PD-1 and anti-CTLA-4 mAbs is closely related to IDO (101). Adenosine exerts an inhibitory role and directly impedes effector T cell function by activating the adenosine receptor A2aR (102). Indirect destruction of effector T cell function is induced by increased Treg function, decreased APC activation, and MDSC induction (103). Moreover, VEGF-A is generated by tumor cells in the TME. In addition to its proangiogenic properties, VEGF-A inhibits dendritic cell maturation, increases the accumulation of MDSCs and induces Tregs, all of which contribute to the formation of an immunosuppressive TME milieu (104, 105). Coexpression of various IRs is increased by VEGF-A in a VEGF-R2- and NFAT-dependent manner, including PD-1, CTLA-4, and Tim-3 (106). In addition, TGF-β is involved in T cell dysfunction in cancer (107). TGF-β inhibits antitumor immunity and shapes the TME by impeding T cell infiltration (108).

IL-35 is expressed by activated Treg cells and regulatory B cells (109–111). A recent study showed that Treg-derived IL-35 could promote T cell exhaustion (112). The underlying mechanism was direct regulation of Blimp1 expression in CD8^+^ T cells, increased IR expression and restricted differentiation of central memory CD8^+^ T cells (112). In addition, neutralization of IL-35 promotes CD8^+^ TIL proliferation and production of inflammatory cytokines (113). Neutralization of IL-35 also decreases tumor growth in a mouse tumor model (113). These results indicate that IL-35 favors the immunosuppressive TME.

### Metabolic Pathways in the TME

The activation of T cells and exertion of antitumor immunity depend on some common metabolic pathways, such as aerobic glycolysis, amino acid metabolism, glutaminolysis and *de novo* fatty acid synthesis (114). These metabolic pathways are also important for cancer cell proliferation and survival. Hence, within the TME, T cells compete with cancer cells to obtain adequate nutrients (Figure 3d).

Recently, some studies have demonstrated that cancer cells compete with TILs to acquire the essential glucose, which results in less availability of glucose to T cells (115). The reduction or deprivation of glucose induces T cell dysfunction and impairs the immune response (116, 117). Therefore, restraining the metabolic activity of tumor cells may have potential value in increasing the amount of glucose available to T cells, thereby improving the antitumor activity of T cells. Moreover, glycolysis is required for T cell maturation, expansion and effector function (114, 118). The CD28 signaling pathway can facilitate the transition of T cell metabolism to glycolysis. Therefore, both PD-1 and CTLA-4 can restrict T cell glycolysis by interfering with CD28 signaling. Compared to CTLA-4, PD-1 can also induce the fatty acid β-oxidation (FAO) rate-limiting enzyme carnitine palmitoyl transferase (Cpt1a) to simultaneously promote FAO (119). Notably, the role of PD-1 in inducing FAO relies on the simultaneous inhibition of the PI3K/AKT and MEK/ERK signaling pathways, which regulate glucose metabolism (119). Blocking PD-1 or CTLA-4 restores the glycolytic ability of TILs in a mouse model, which promotes glycolysis in T cells (115). It is noteworthy that PD-L1 derived from tumor cells can facilitate tumor glycolysis through AKT/mTOR signaling (115). Thus, blocking the PD-1/PD-L1 axis can lead to a range of synergistic antitumor effects: restoring tumor-specific T cell function, reducing tumor glycolysis, and improving glucose availability to T cells in the TME.

Of course, certain amino acids, including arginine, glutamine and tryptophan, are also important nutrients that stimulate T cell activation and promote cancer cell proliferation and survival. For instance, arginine is a nutrient necessary for T cell activation and proliferation (120). TAMs and MDSCs express arginase 1, which decomposes and reduces arginine in the TME, and results in impaired effector T cell function (121–123). Interestingly, for T cell activation and differentiation, glutamine is essential (124). However, cancer cells exert a strong intake of glutamine, which is conducive to tumor development (125). It is well known that the rate-limiting enzyme for glutamine decomposition is glutaminase. In a mouse model, targeting glutaminase is effective in controlling tumor occurrence (126). Therefore, we can infer that excessive glutaminolysis in the TME is due to cancer cells, which restricts the utilization of glutamine by T cells and thereby promotes T cell dysfunction. Moreover, because tumor cells and suppressive immune cells secrete the tryptophan metabolism enzyme IDO, tryptophan is catabolized into kynurenine, an immunosuppressive metabolite (127). Accumulated kynurenine facilitates regulatory T cell production (128). Collectively, these results indicate that in the TME, T cells can effectively exert antitumor immunity, which is inseparable from some important nutrients. These essential nutrients may be controlled by cancer cells and immune regulatory cells.

In addition to nutrients, various metabolites are also involved in T cell dysfunction, such as lactic acid, low pH, and hypoxia (Figure 3d). Tumor-derived lactic acid can induce apoptosis in naive T cells, which may support tumor immune escape, due to the loss of the 200 kDa FAK family interacting protein (129). Lactic acid induces pH alterations and a loss of cytosolic NAD^+^ regeneration, which further limits T cell activity and cytokine production (130). Thus, neutralizing acidic components in the TME may contribute to enhancing T cell antitumor immunity (131). Notably, hypoxia is another metabolic parameter in the TME due to rapid tumor growth. Different studies have shown that hypoxia improves or impairs T cell immune responses. In murine chronic infections and tumor models, results indicate that hypoxia can enhance the cytotoxic function of T cells as well as lead to more effective viral and tumor control (132, 133). By contrast, increased expression and activity of the transcription factor hypoxia-inducible factor 1α (HIF-1α) is induced by hypoxia, which increases the expression of inhibitory receptors on T cells and reduces T cell effector function (132, 134).

### Epigenetic Imprinting of T Cell Dysfunction

The epigenetic imprinting of dysfunctional T cells differs from that of effector/memory T cells (Figure 3e). Normally, PDCD1 (encoding PD-1) is temporarily demethylated in activated T cells, after which normal methylation levels are restored. In chronic viral infections, results indicate that the PDCD1 demethylation levels persist in activated T cells, leading to long-term expression of PD-1 and T cell dysfunction (135–137). Even after the virus is cleared to levels that cannot be detected by current technologies, this demethylation pattern of the PDCD1 locus is still sustained, suggesting that this epigenetic program has been fixed. Interestingly, one study reported the presence of two T cell clones with different affinities in Melan-A-specific TILs in human melanoma (138). Low avidity T cell clones, due to the sustained methylation of the PDCD1 promoter, do not express PD-1 even when stimulated by TCR. Conversely, antigen-specific T cells with high functional avidity express PD-1. These phenomena suggest a relationship among expression of PD-1, modification of the PDCD1 locus and intensity of TCR signaling (138). Aside from the continued demethylation of the PDCD1 locus, dysfunctional T cells gradually undergo *de novo* methylation of genes involved in effector functions during persistent infections and tumor progression, limiting T cell expansion and clonal diversity (139). This cannot be reversed by PD-1 blockade. However, during PD-1 blockade, blocking dysfunction-related methylation imprinting has a synergistic effect in a murine tumor model, as manifested by increased T cell immune responses and tumor control (139).

In addition, unique changes in chromatin accessibility occur in dysfunctional T cells. For example, in chronic LCMV infection, additional accessible regions of PDCD1 appeared in dysfunctional T cells (140). However, some open regions of the IFNG locus were present in functional T cells and were absent in dysfunctional T cells. It is worth noting that after anti-PD-1 treatment, these accessible areas specific to the dysfunctional state did not significantly change, showing that the epigenetic program was only minimally remodeled (141). Similarly, tumor antigen-specific and bystander T cells were adoptively transferred in a murine tumor model (142). Gene expression and chromatin accessibility were then compared. The results showed that only tumor-specific T cells were characterized by dysfunction and exhibited increased effector functions after anti-PD-L1 therapy, with little change in chromatin accessibility (142).

Moreover, epigenetic imprinting begins in the early stages of dysfunction. Many changes in dysfunctional T cells in advanced tumors of mouse liver cancer models are also present in T cells from precancerous lesions (7). Two different chromatin states can mirror the process of moving from mild dysfunction in early tumor lesions to more severe dysfunction in late-stage tumors. On the one hand, the chromatin state of early dysfunctional TILs shows partial plasticity and can be reprogrammed by anti-PD-1 therapy. On the other hand, the advanced state is fixed and cannot be changed by anti-PD-1 therapy (143). It is noteworthy that most of the overlap exists between the chromatin accessibility state of PD-1^hi^ CD8^+^ TILs from human non-small-cell lung cancer and the chromatin accessibility state of late TILs in mice. Thus, PD-1/PD-L1 blockade in human tumors may only cause transcriptional rewiring rather than altering the chromatin accessibility pattern itself.

### Transcriptional Regulation of T Cell Dysfunction

Transcriptional regulation of T cell dysfunction involves changes in the expression patterns and transcriptional connection of some important transcription factors. Some transcription factors are expressed in both functional Teff and dysfunctional T cells (Figure 3f). However, in dysfunctional T cells, these transcription factors are linked to different genes and transcriptional loops, showing activity in specific contexts (144, 145).

T-bet and Eomes, as T-box family transcription factors, play a crucial role in the development of effector and memory CD8^+^ T cells (146, 147). In acute infection, T-bet promotes the formation of Teff cells and the development of KLRG-1^+^ terminal Teff cells, while Eomes promotes the expression of IL-15Rβ and the development of memory T cells in a homeostatic manner (2, 144, 147–149). However, in chronic infection, the role of T-bet and Eomes is different from their function in Teff and Tmem cells (145). For example, the formation of a dysfunctional T cell population requires the participation of both T-bet and Eomes. And if either transcription factor is genetically deleted, the dysfunctional T cell population cannot form (144). Furthermore, due to its potential ability to inhibit the transcription of the IR genes (e.g., Pdcd1 encoding PD-1), upregulated expression of T-bet favors the formation of progenitor PD-1^int^ Eomes^lo^ Tex subpopulation (144). As for Eomes, its high expression in the terminal Tex subpopulation differs from that in the self-renewal Tmem cells in acute infection (144). In view of this, transcriptional network analysis is performed, and it has been shown that Eomes participates in almost completely different transcription networks in functional Teff and Tmem cells compared with Tex cells (145). Therefore, the functions of T-bet and Eomes are rewiring in Tex cells.

Other transcription factors are also involved in T cell dysfunction. Foxo1, Blimp-1, NFAT, IRF-4, and BATF can promote or antagonize T cell dysfunction by modulating dysfunction or effector-specific genes, respectively (12, 150–154). The expression level of these transcription factors directly affects their capability of driving distinct transcriptional programs. For instance, dysfunctional T cells upregulate Foxo1 and Blimp-1, which act as positive regulators of T cell dysfunction (150, 152, 155). Also, metabolic-related transcription factors, including hypoxia-inducible factor (HIFs) and von Hippel-Lindau (VHL) complexes, may promote T cell dysfunction in some cases (156). In addition, recent research has found that TOX, as a new transcription factor, promotes T cell dysfunction in cancer (157–159).

## Heterogeneity in Dysfunctional T Cells

Dysfunctional T cells are heterogeneous. Firstly, PD-1 and CD44 are utilized to distinguish subpopulations of dysfunctional T cells with various biological functions. Studies have revealed that PD-1^int^CD44^hi^ T cells had a lower degree of dysfunction than PD-1^hi^CD44^int^ T cells, which may be partially explained by lower coexpression of IRs (9). Furthermore, the PD-1^int^ T cells can produce a therapeutic response to PD-1 blockade. Conversely, PD-1^hi^CD44^int^ T cells are in a terminally dysfunctional state and have no therapeutic response to immune checkpoint blockade (9). Subsequent studies have also shown that the PD-1^int^CD44^hi^ T cell subpopulation is mainly composed of T-bet^hi^ and Eomes^lo^ cells, and as a progenitor cell population, can persistently generate PD-1^hi^CD44^int^Eomes^hi^ T cell population with terminal dysfunction state (144).

In addition, the understanding of dysfunctional T cell heterogeneity is further improved due to the recognized role of CXCR5 and the transcription factor Tcf-1. The CXCR5^+^Tcf-1^+^Tim-3^−^ T cell subpopulation can respond to PD-1 blockade and produce a more terminal CXCR5^−^Tcf-1^−^Tim-3^+^ T cell subpopulation (160). Interestingly, there is no difference in the expression of the transcription factors T-bet and Eomes between the two subpopulations (160). These results may reveal the heterogeneity in the original PD-1^int^ progenitor cell populations.

Therefore, the heterogeneity of dysfunctional T cells is complex. Identifying progenitor populations that respond to checkpoint blockade may be more beneficial to the development of cancer immunotherapy and to cancer patients.

## Reversal of Dysfunctional T Cells by Immunotherapy

### Single or Combined Immune Checkpoint Blockade

Immunotherapy for treating tumor-induced T cell dysfunction has provided hope to patients with cancer. Currently, immunotherapy has focused on blocking immune checkpoints in patients with various solid and hematological tumors (161–164). The most commonly targeted immune checkpoints are PD-1 and CTLA-4. Generally, PD-1 impedes T cell function by interfering with T cell receptor (TCR) signaling, whereas CTLA-4 impedes T cell function by competing with the costimulatory molecule CD28 to bind to CD80/CD86 (165, 166). Since Treg cells also express CTLA-4, antibodies targeting CTLA-4 can inhibit Treg cell function or enhance antitumor immunity by selectively eliminating Treg cells in a mouse model (62). The CTLA-4 targeting agent, ipilimumab, was the first immune checkpoint inhibitor. With the use of ipilimumab, overall survival was significantly increased in patients with advanced melanoma (167, 168). Of note, in patients with melanoma treated with anti-CTLA-4, the melanoma-reactive CD8^+^ T cell response in peripheral blood was found to be significantly increased (169). This result suggests that enhanced T cell priming also plays a role. Also, PD-1 pathway blockade can effectively reverse dysfunctional T cells and increase antitumor activity in patients with various cancers, especially in viral infection-driven cancers or carcinogen-induced cancers (170–174). In addition, as mentioned above, recent studies have revealed that the PD-1/PD-L1 axis inhibits T cell function through the inactivation of CD28 signaling instead of TCR signaling (48). In a mouse model, conditional knockout of CD28 abolished the effects of PD-1 blockade (49), suggesting that the CD28/B7 pathway may act as an important role in the efficacy of anti-PD-1 therapy. Currently, five anti-PD-1 or anti-PD-L1 antibodies have been approved by the FDA for the treatment of a variety of cancers, including hepatocellular carcinoma, renal cell carcinoma and Hodgkin's disease (175).

Moreover, compared with monotherapy, the combined use of anti-CTLA-4 and anti-PD-1 has demonstrated prolonged progression-free survival and significant tumor regression in clinical trials of cancer patients (176, 177). Also, PD-1 blockade combined with other checkpoint blockades, such as Tim-3, LAG-3, or TIGIT, significantly reverses T cell dysfunction and enhances antitumor immunity in patients with cancer, compared to individual checkpoint blockade (25, 26, 178, 179). At present, humanized anti-TIM3 (TSR-022), anti-LAG3 (MK-4280), and anti-TIGIT (BMS-986207) antibodies against various cancers are under clinical trials. However, the underlying precise mechanisms to explain the efficacy of immune checkpoint blockade have not been fully described.

### Immune Checkpoint Blockade in Combination With Other Immunotherapies

The combination of inhibitory receptor blockade and costimulatory molecule targeting exerts a synergistic effect in re-activating tumor-specific T cells. CD137 (or 4-1BB), a member of the TNFR family, is expressed on activated T cells (180). In a murine model, the combination of agonistic anti-CD137 antibodies and PD-1 blockade effectively controls tumor growth (181). This combination therapy also improves the T cell response to tumor antigens and promotes effector/memory CD8^+^ T cell formation (182). Another member of the TNFR family, OX40, is primarily expressed on activated CD4^+^ T cells. And its expression on CD8^+^ T cells is at a lower level upon TCR triggering (183). The research results show that OX40 agonists play an antitumor immunity-promoting role in immunogenic mouse tumor models, but do not play an effective role in tumor control in a poor immunogenic tumor model (184, 185). Strikingly, in a vaccination setting, the addition of PD-1 blockade to OX40 agonists virtually eliminates the antitumor effect of OX40 monotherapy due to the reduced TIL infiltration and enhanced cell death of tumor-reactive CD8^+^ T cells (186). This effect deserves close attention because it is uncertain whether a similar harmful effect will occur in human cancers. Also, this phenomenon underscores the importance of the timing of different immunotherapeutic interventions to avoid the negative effects of T cell overstimulation.

Furthermore, anti-CTLA-4 therapy in combination with anti-VEGF antibodies has been shown to increase antitumor immune responses and achieve encouraging clinical results in patients with metastatic melanoma (187). Moreover, in mice with melanoma, researchers have demonstrated that the use of PPARα agonists can promote fatty acid catabolism in vaccine-induced CD8^+^ TILs. The addition of anti-PD-1 antibodies obviously enhanced the antitumor efficacy (188).

Therefore, future research should focus on how combination therapies should be applied and which dysfunctional states may be best for them.

### CAR-T Cell Therapy

A CAR-T cell is genetically modified to express an antigen-specific, non-MHC restricted receptor, which consists of the single-chain variable fragment (scFv) of an antibody, a transmembrane domain and an intracellular signaling domain (189, 190). Thus far, CAR-T cell therapy has achieved great success in treating hematological malignancies (191, 192). However, some patients still lack response and relapse after receiving CAR-T treatment, possibly due to the poor proliferative capacity and the short duration of T cells. Therefore, a transcriptomic analysis was conducted to compare T cells from chronic lymphocytic leukemia (CLL) responders and non-responders after CAR-T treatment (193). The results showed that T cells from non-responders upregulated pathways associated with exhaustion and apoptosis compared to T cells from responders (193). Inhibitory receptors were also upregulated on these CAR-T cells, indicating that these inhibitory molecules may result in dysfunction and poor persistence of CAR-T cells (194, 195).

Recently, CRISPR-Cas9 technology has been applied to knock out IR itself, and studies have been conducted on PD-1 and LAG-3 in CD19-BBζ CAR-T cells (196, 197). In mouse xenograft models, knockout of IR-derived CAR-T cells effectively eradicated tumors (196, 197). This method has also been applied to solid tumors. CRISPR-Cas9 was utilized to knock out PD-1 in CD133-specific CAR-T cells. In a mouse glioma model, CD133-specific CAR-T cells with PD-1 knockout enhanced the control of tumor growth compared to control CD133-CAR-T cells (198). Based on the encouraging results obtained above, CAR-T cells edited by CRISPR-Cas9 have entered clinical trials. For example, CD19-specific CAR-T cells with PD-1 knockout are being investigated in PhaseIclinical trials (NCT03298828).

Additionally, CAR-T cells are designed to secrete immune checkpoint antibodies themselves. In CD19^+^ lung cancer xenograft models, CAR-T cells have been engineered to secrete anti-PD-1 antibodies and significantly improved antitumor activity, manifested by enhanced T cell proliferation, increased cytotoxicity, and prolonged overall survival (199). Compared with standard CAR-T cells, anti-CAIX CAR-T cells secreting anti-PD-L1 antibodies significantly increase antitumor activity, as evidenced by enhanced cytokine production and immune cell recruitment and the significant reduction in tumor size in the humanized ccRCC mouse model (200). Whether these CARs are likely to succeed in the body is still unknown. Currently, CAR-T cells, which are designed to secrete PD-1, CTLA-4 or PD-L1 antibodies, have entered clinical trials for cancers expressing MUC1, EGFR, EGFRVIII, and mesothelin (201).

In addition, other strategies have been used to enhance CAR-T cell function in TME, including inhibition of inhibitory soluble molecules such as IDO, adenosine and VEGF, and protection from immunosuppression of non-tumor cells, such as MDSCs and TAMs. Interestingly, the combination of EGFRVIIICAR-T cells with the blockade of IDO1 significantly reduces tumor growth in a xenograft colon cancer model (202). In HER2 CAR-T cells, blockade of adenosine 2A receptor enhances CAR- T cell activation and cytokine production, which contributes to the improved antitumor efficacy of CAR-T cells (203). The addition of PD-1 blockade further enhanced T cell immunity (203). Also, VEGF blockade has achieved success in solid tumors including melanoma, and VEGF-targeted CAR-T cells have achieved good results in a variety of preclinical solid tumor models (187, 204, 205).

## Challenges and Possible Solutions

Resistance to therapy is a major challenge in cancer immunotherapy. Defects involved in IFNγ signaling and antigen presentation pathways, as tumor-intrinsic alterations, can lead to resistance to cancer immunotherapy, which explains why immunotherapy is not effective in some cancer patients (206, 207). In melanoma patients treated with PD-1 blockade, for example, the acquired resistance is attributed to the inactivation of IFNγ signaling and defects in the antigen presentation mechanism (206).

Moreover, alterations in the functional state of TILs are also among the reasons for resistance to immunotherapy (208). Interestingly, after adoptive transfer of MART-1 TCR-transduced T cells in advanced melanoma patients, the T cell functional phenotype was altered during relapse, which was characterized by a complete loss of initial cytotoxic activity or an acquired inflammatory cytokine secretion but lack of cytotoxicity (209). Therefore, to assess whether changes in the dysfunctional state are also critical in mediating the immunotherapeutic resistance of cancer patients, it will be of value to compare monotherapy and combination therapy at the beginning of treatment and at the moment of resistance.

In addition, reactivation of treatment-induced tumor-specific T cells may unexpectedly promote the further progression of T cell dysfunction. By comparing longitudinal samples of colon cancer models in both immunocompetent and immunodeficient mice, we found two important tumor escape mechanisms involving the accumulation of immunosuppressive cell populations (e.g., Tregs) and enhanced expression of multiple inhibitory receptors on T cells, which contribute to the deterioration of T cell function (210). Given this, Chauvin et al. discovered that, after PD-1 blockade *in vitro*, TIGIT expression was increased on TA-specific CD8^+^ T cells (25). Dual TIGIT and PD-1 blockade could promote the effector function of CD8^+^ T cells. Similarly, Tim-3 was upregulated on TILs in a PI3K/AKT-dependent manner upon PD-1 blockade. Notably, the combined blockade of the two receptors significantly improved antitumor immunity (211). In a mouse lung cancer model, several other immune checkpoints, such as CTLA-4, Tim-3, and LAG-3, were also found to be upregulated upon resistance to PD-1 blockade (212). Therefore, upregulation of other immune checkpoints also provides a potential resistance mechanism after anti-PD-1 treatment.

## Conclusions

Dysfunctional T cells have various cellular states and different characteristics in cancer. Both persistent TCR triggering and T-cell internal and external factors can affect the fate of dysfunctional T cells. Although the characteristics of T cell dysfunction in cancer are similar to those of T cell dysfunction in chronic viral infection, there are various factors in the complicated TME that promote the process of immunosuppression, which can further shape T cell dysfunction in cancer. Gradually, it is conceivable that dysfunctional T cells in cancer differ from those found in chronic infection sites. However, heterogeneous dysfunctional T cell states still exist among different tumors and even within tumors. To reverse dysfunctional T cells and restore antitumor immunity, immune checkpoint blockade and related combination therapies have been applied. However, some challenges still remain to be solved, such as how to maintain long-lasting efficacy and how to choose the best combination therapy. Moreover, it is essential to understand the processes that drive and maintain these different dysfunctional T cell states. Continued advances in technology will help achieve this goal, supporting the development of personalized strategies for targeting dysfunctional T cells to achieve precise treatment for cancer patients.

## Author Contributions

AX conceived and wrote the manuscript and prepared the figures. YZ and JX contributed to performing the literature collection. TY and X-JL directed and approved the manuscript. All authors declare no conflict of interest and gave the final approval of the manuscript submission.

### Conflict of Interest Statement

The authors declare that the research was conducted in the absence of any commercial or financial relationships that could be construed as a potential conflict of interest.
